# Supplementary figures and images for: Modeling the sequence dependence of differential antibody binding in the immune response to infectious disease
Source: PLoS Comput Biol. 2023 Jun 20;19(6):e1010773. doi: 10.1371/journal.pcbi.1010773 (PMC10313026; doi:10.1371/journal.pcbi.1010773)

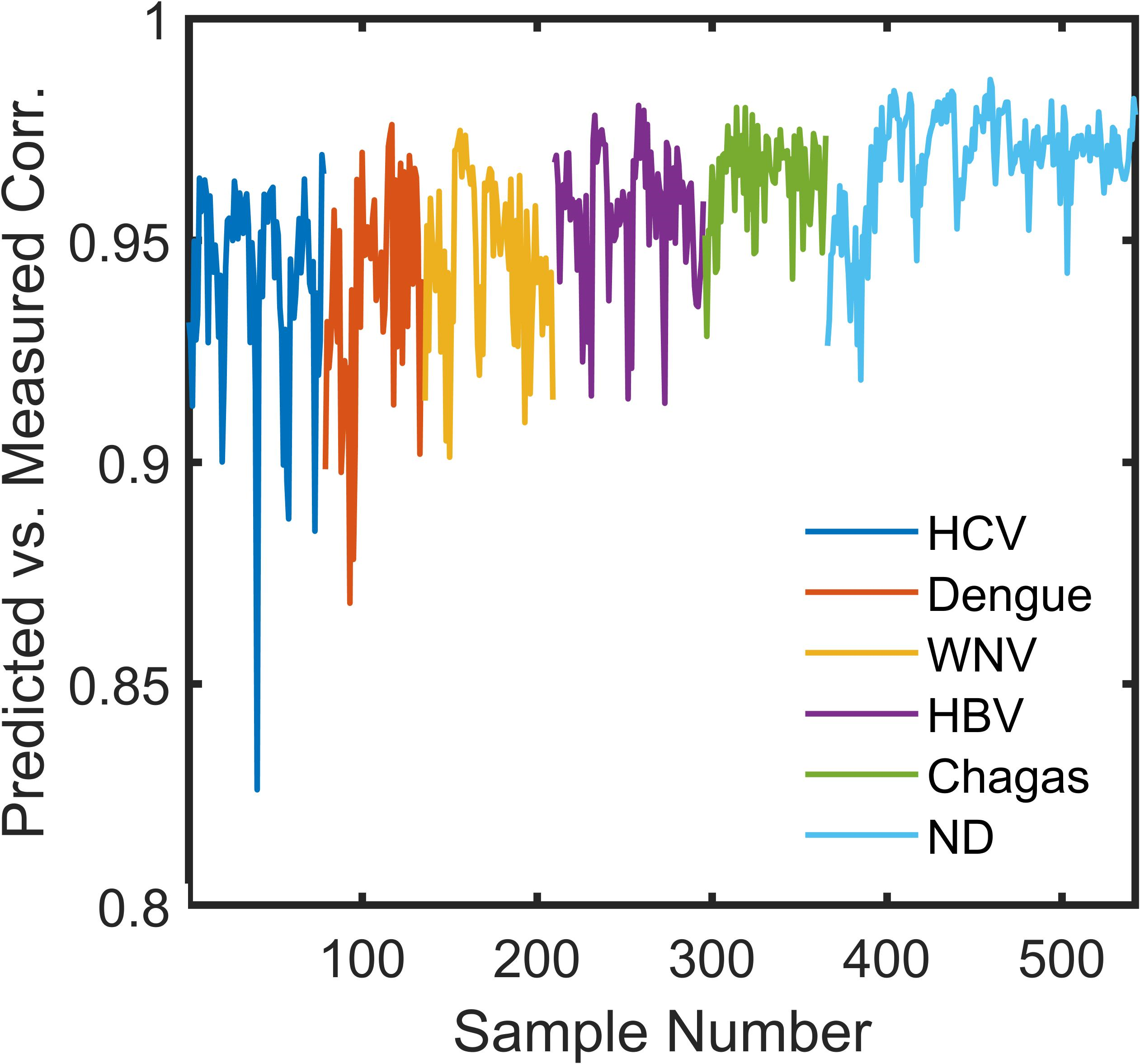

Supplement: S1 Fig — (TIF) [file pcbi.1010773.s001.tif]

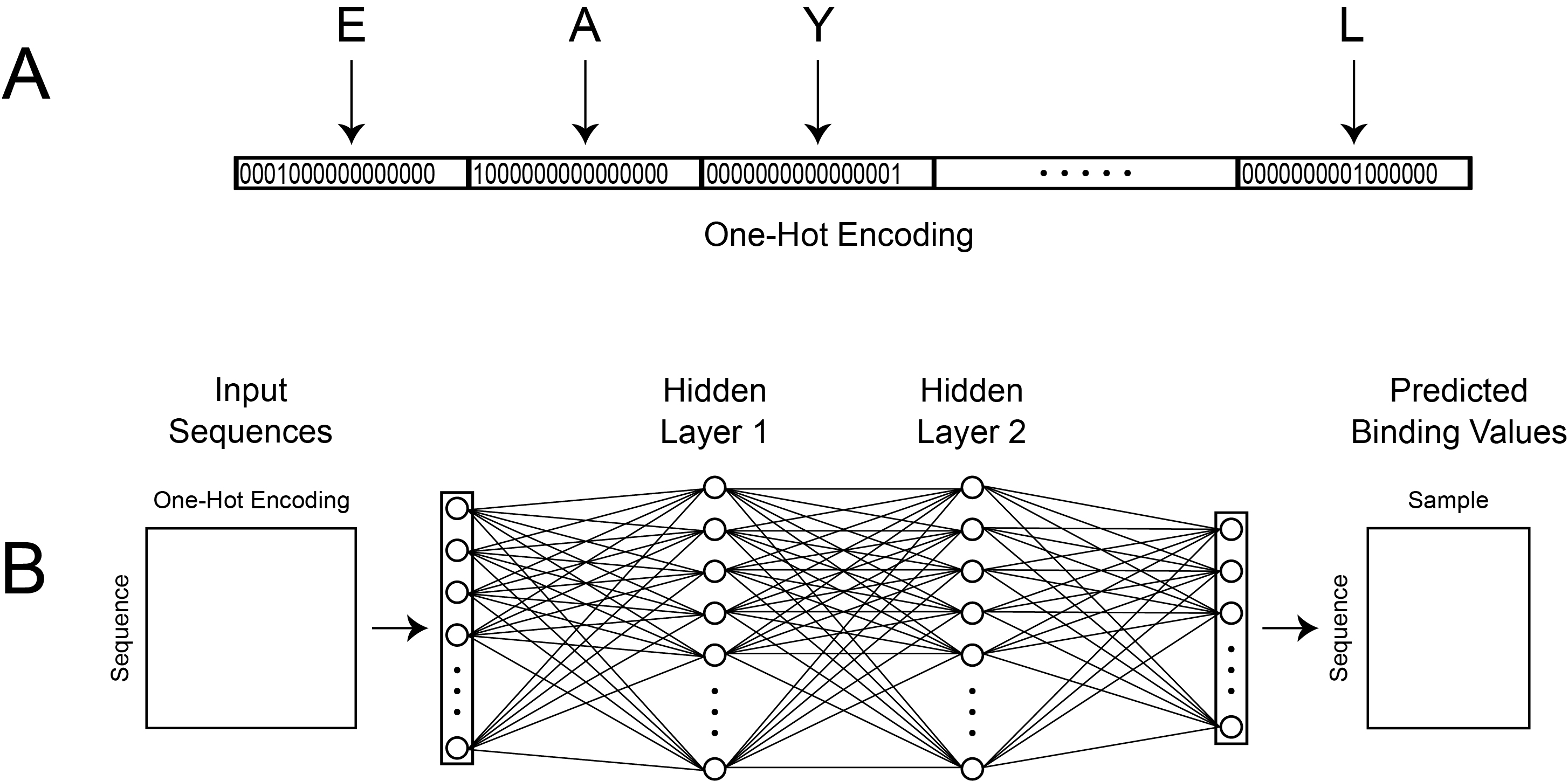

Supplement: S2 Fig — (TIF) [file pcbi.1010773.s002.tif]

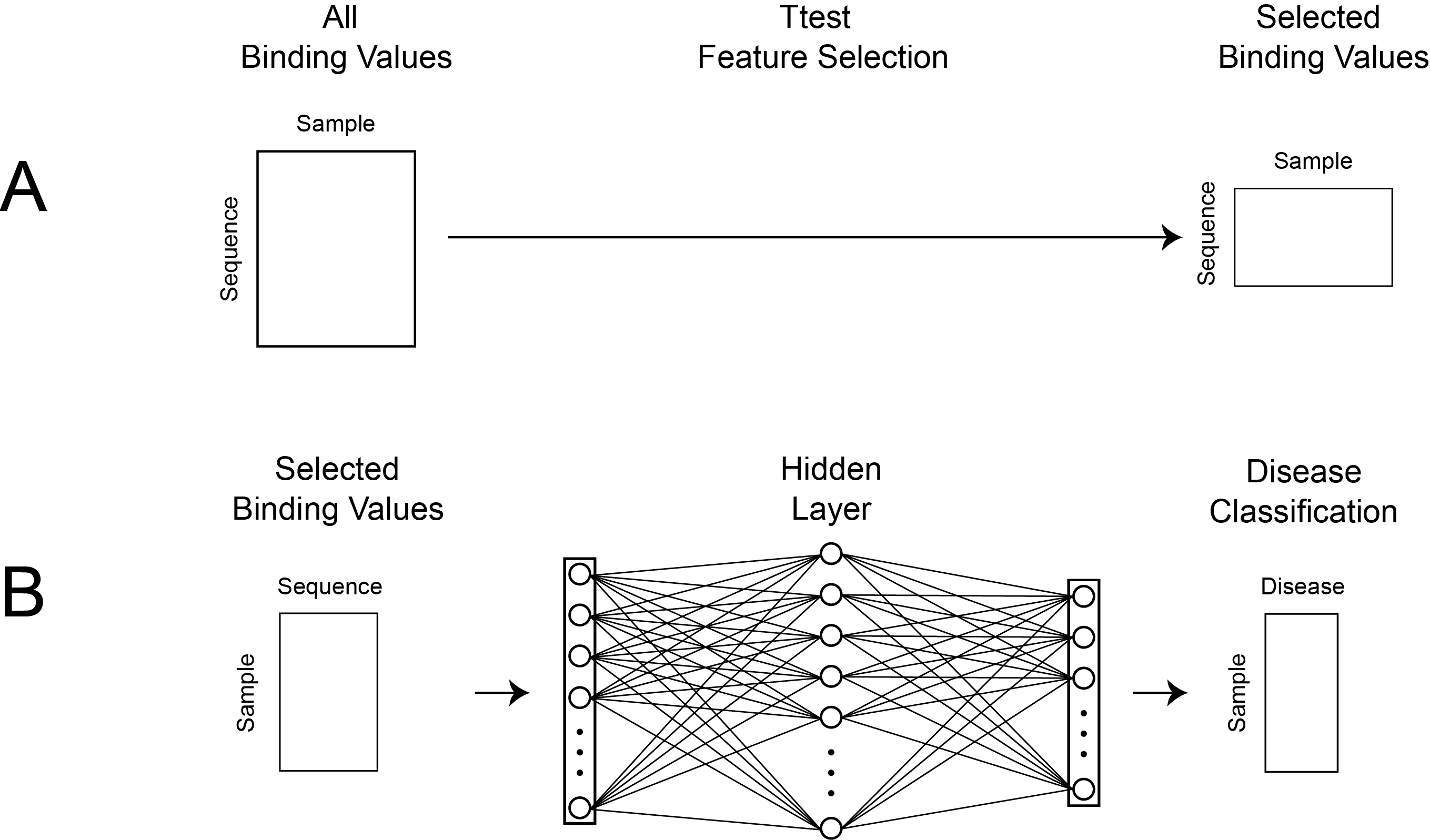

Supplement: S3 Fig — (TIF) [file pcbi.1010773.s003.tif]

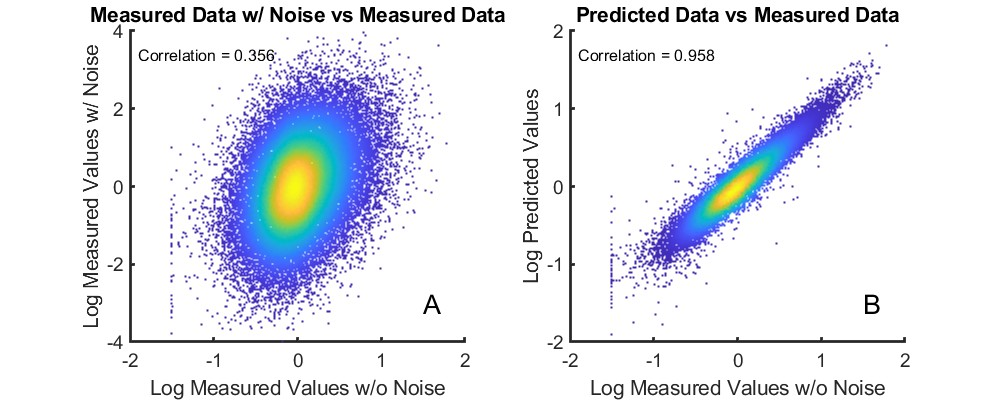

Supplement: S4 Fig — (A) a scatter plot of the log10 of the measured data values with sigma = 1 gaussian noise added vs. the log10 measured data values without noise added. (B) in this scatter plot, the Y-axis is log10 of the binding values predicted from a sequence-binding neural network model generated using the measured data values with sigma = 1 gaussian noise added and the X-axis is again the log10 of the measured values without noise. The function dscatter was used to generate a density-colored scatter plot (see the description of Fig 2B ref. 52 in the text). The neural network sequence-binding relationship effectively removed the sequence independent noise that was added to the data. (TIF) [file pcbi.1010773.s004.tif]
